# Assessment of Fetal Kidney Growth and Birth Weight in an Indigenous Australian Cohort

**DOI:** 10.3389/fphys.2017.01129

**Published:** 2018-01-09

**Authors:** Christopher J. Diehm, Eugenie R. Lumbers, Loretta Weatherall, Lyniece Keogh, Sandra Eades, Alex Brown, Roger Smith, Vanessa Johnson, Kirsty G. Pringle, Kym M. Rae

**Affiliations:** ^1^Gomeroi Gaaynggal Centre, Faculty of Health and Medicine, University of Newcastle, Callaghan, NSW, Australia; ^2^Priority Research Centre for Reproductive Sciences, University of Newcastle, Newcastle, NSW, Australia; ^3^Department of Rural Health, University of Newcastle, Tamworth, NSW, Australia; ^4^Faculty of Health and Medicine, School of Biomedical Sciences and Pharmacy, University of Newcastle, Callaghan, NSW, Australia; ^5^Heart Failure Research Group, Baker IDI Heart and Diabetes Institute, Melbourne, VIC, Australia; ^6^Aboriginal Research Unit, South Australian Health and Medical Research Institute, Adelaide, SA, Australia

**Keywords:** indigenous health, fetus, kidney, nephron, ultrasound, smoking

## Abstract

**Introduction:** Indigenous Australians experience higher rates of renal disease and hypertension than non-Indigenous Australians. Low birth weight is recognized as a contributing factor in chronic disease and has been shown to increase the risk of renal failure in adulthood. A smaller kidney volume with fewer nephrons places an individual at risk of hypertension and renal failure. Indigenous Australians have fewer nephrons than non-Indigenous Australians. In this study, intrauterine fetal and kidney growth were evaluated in 174 Indigenous Australian babies throughout gestation in order to record and evaluate fetal growth and kidney size, within a population that is at high risk for chronic illness.

**Methods:** Pregnant women that identified as Indigenous, or non-Indigenous women that were pregnant with a partner who identified as an Indigenous Australian were eligible to participate. Maternal history, smoking status, blood and urine samples and fetal ultrasounds were collected throughout pregnancy. Fetal kidney measurements were collected using ultrasound. Statistical analysis was performed using the Stata 14.1 software package.

**Results:** 15.2% of babies were born prematurely. 44% of the mothers reported smoking in pregnancy. The median birth weight of this cohort was 3,240 g. Male fetuses had higher kidney to body weight ratios than female fetuses (*P* = 0.02). The birth weights of term neonates whose mothers smoked during pregnancy were lower (327 g, *P* < 0.001) than the birth weights of term babies from non-smoking mothers. The kidney volumes of babies whose mothers smoked were also smaller (*P* = 0.02), but were in proportion to body weight.

**Conclusion:** In this cohort of Indigenous women smoking was associated with both increased number of preterm births and with a reduction in birth weights, even of term infants. Since kidney volume is a surrogate measure of nephron number and nephrogenesis is complete at birth, babies whose mothers smoked during pregnancy must have fewer nephrons than those from non-smoking mothers. Previous studies have shown that glomerular filtration rate is not related to birth weight, thus infants with smaller kidney volumes are hyperfiltering from birth and therefore are likely to be more susceptible to early onset renal disease in later life.

## Introduction

In the 1980's David Barker postulated that the intrauterine environment could influence the future health of an individual (Barker et al., 1989a). He derived this conclusion from a cohort study that revealed an association between low birth weight and the onset of chronic diseases in adult life (Barker et al., 1989a,b, 1993). This is now known as the developmental origins of health and disease (DOHaD) hypothesis (Sinclair et al., 2007; Vignini et al., 2012; Heindel and Vandenberg, 2015).

It is well-established that the Australian Aboriginal and Torres Strait Islander (Indigenous) population have much higher rates of renal disease, hypertension and cardiovascular disease than non-Indigenous Australians (Australian Institute of Health and Welfare, 2011; Australian Indigenous HealthInfoNet, 2014). Hoy et al. found that in a population of Indigenous men and women, aged 20–38 years of age, from the Northern Territory of Australia, lower birth weights predisposed to albuminuria in adult life and amplified the levels of albuminuria that accompanied compounding factors such as age, elevated body mass index (BMI) and blood pressure. Given that overt albuminuria indicates renal dysfunction, and predicts renal insufficiency, it was concluded that being born with low birth weight predisposed an individual to early onset renal failure (Hoy et al., 1999).

Since nephrogenesis in the human fetus is complete before term, fewer layers of nephrons are created if intrauterine growth is restricted (Hinchcliffe et al., 1992) so that growth restricted neonates have fewer nephrons (Koike et al., 2017). There is a correlation between high blood pressure (Systolic BP > 150 mmHg) and the number of nephrons an individual is endowed with (Hayman et al., 1939). Hayman et al. reported that of those investigated; only 15% of subjects with more than 1,000,000 glomeruli had hypertension before death. However, this rose to 33% in those with 700,000–1,000,000 glomeruli and rose further again to 100% of those individuals with less than 700,000 glomeruli (Hayman et al., 1939). In 2003, Keller et al. conducted a post-mortem assessment of the kidneys of 20 subjects who had died in accidents. Ten of these subjects had a history of hypertension, while 10 did not. It was found that the subjects with a history of hypertension had significantly fewer glomeruli per kidney (702,379 vs. 1,429,200) than those without hypertension (Keller et al., 2003). A study of Indigenous Australians based in the Northern Territory of Australia showed that they had 30% fewer glomeruli than non-Indigenous Australians. This equated with an estimated 404,000 fewer glomeruli per person. Among the Indigenous Australians included in this study, those with hypertension had 30% (500,000) fewer glomeruli than those that did not have hypertension. With regard to glomerular volume, the Indigenous Australians in this study had a 27% larger glomerular volume, which is likely explained by compensatory hypertrophy in response to reduced total glomerular number (Hoy et al., 2006).

Brenner et al. (1988) proposed that either a congenital or programmed reduction in the total number of nephrons within a kidney was related to a higher risk of hypertension and kidney disease (Brenner et al., 1988). The proposal was that the fewer the number of nephrons, the less the surface area available for glomerular ultrafiltration. Since glomerular filtration rate is determined by body surface area and all nephrons are formed prior to birth, it follows that the fewer the nephrons an individual possesses, the greater the filtration rate per nephron (i.e., single nephron filtration rate). That is, there is hyper-filtration leading to glomerular damage. This offers an explanation as to why populations with a smaller number of nephrons within their kidneys are more susceptible to compensatory hypertrophy and subsequently kidney disease (Brenner et al., 1988; Valerie and Brenner, 2010).

Kidney volume has been suggested as a surrogate measure of nephron number (Nyengaard and Bendtsen, 1992). Hinchcliffe et al. showed that low birth weight babies had fewer nephrons and there was a strong correlation between nephron number and renal volume in neonates (Hinchcliffe et al., 1992). Low birth weight babies have smaller kidney volumes, yet by 6 days after birth they have similar glomerular filtration rates to appropriately grown babies (Kandasamy et al., 2013). Previous research by Kandasamy et al. also showed that Indigenous Australian neonates (both term and preterm) had smaller kidney volumes than non-Indigenous neonates, and therefore fewer nephrons. These Indigenous neonates had similar estimated glomerular filtration rates, which must have been achieved by higher single nephron filtration rates (Kandasamy et al., 2014). That is, hyperfiltration was occurring in these neonates from birth onwards. Since hyperfiltration increases the risk of glomerular damage, Indigenous neonates are at a higher risk of renal disease in adult life (Valerie and Brenner, 2010; Kandasamy et al., 2014).

The aim of this study was to measure fetal kidney growth *in utero* and birth weight in an Indigenous population located in northwest New South Wales, Australia. Given that the rate of chronic kidney disease is 3.7 times higher among Indigenous Australians compared with non-Indigenous Australians (Australian Institute of Health and Welfare., 2015), and as outlined above, *in utero* conditions critically influence the development of chronic diseases, this study assessed renal development *in utero* by measuring kidney volume and determining if there were any associations between kidney size and other fetal and maternal parameters.

## Methods

### Setting

The Gomeroi gaaynggal study is a prospective longitudinal cohort study involving mothers and their Indigenous Australian fetus/child. The primary site of the study is Tamworth, a rural town in New South Wales, Australia. The study began after a 2 year consultation with the local Indigenous community.

### Ethics

Ethics approval for this research was obtained from the Hunter New England Human Research Ethics Committee (HREC Ref. no. 08/05/21/4.01, NSW HREC Ref. no. HREC/08/HNE/129) and the Aboriginal Health and Medical Research Council of NSW (Ref. no. 654/08).

### Recruitment

Recruitment for the study began in 2010. Indigenous research assistants attended antenatal clinics at Tamworth Rural Referral Hospital to recruit eligible women. Pregnant women that identified as Indigenous, or non-Indigenous women that were pregnant with a partner who identified as an Indigenous Australian were eligible to participate. Participants were able to enroll at any stage of pregnancy. The women provided written informed consent to participate in the study.

### Study design

At each visit maternal samples of blood and urine were collected for analysis. Personally reported smoking status was also recorded. During the visits, obstetric ultrasound imaging was undertaken, which included routine morphology, growth and fetal well-being assessment. Ultrasound examinations were performed using a Philips Cx50 Portable Diagnostic Ultrasound with a 5 MHz transducer. During each ultrasound, the size of the fetal kidneys was measured; length, antero-posterior diameter (thickness) and transverse diameter (width). Renal length was recorded as the greatest longitudinal length measured, while renal thickness and width was the greatest measurement obtained above the hilum. Renal volume was calculated using the formula for an ellipsoid shape (Volume = Length × Transverse Diameter × Antero-posterior Diameter X 0.5233) (Cantraine et al., 1986). Estimated fetal weight was calculated by using the Hadlock equation (Hadlock et al., 1985). Background medical information and birth details, including fetal sex and newborn measurements, were collected from the Tamworth Rural Referral Hospital's Medical Records Department.

We attempted to obtain data from every woman in each trimester of their pregnancy. However, while attempts were made to consult participants at these times, data collection was undertaken at times suitable to the participants.

### Statistics

Statistical Analysis was performed using the Stata 14.1 software package (StataCorp LLC, Texas, USA). The relationships between fetal growth and gestation were derived using non-linear regression. Kruskal Wallis Test was used to assess the differences in gestational age at delivery and renal kidney volume between the smoker and non-smoker groups. One-way ANOVA was used to assess the differences in birth weight and renal parameters. *T*-tests were used to evaluate renal parameters between smoking and non-smoking groups. A *P*-value < 0.05 was considered statistically significant.

## Results

One hundred and seventy four babies were recruited to this study. There were 8 sets of twins; they were excluded from the analyses leaving a total of 158 infants. Ninety-four babies were male (59.5%) and 64 were female (40.5%). The median gestational age at delivery was 39.1 weeks; age at delivery of males was 39.1 weeks and for females was 39.3 weeks. 134 babies were born at term (>37 weeks), and 24 of the cohort (15.2%) were born prematurely with a median age at birth of 35.3 weeks (IQR = 2 weeks).

**Figure 1:**
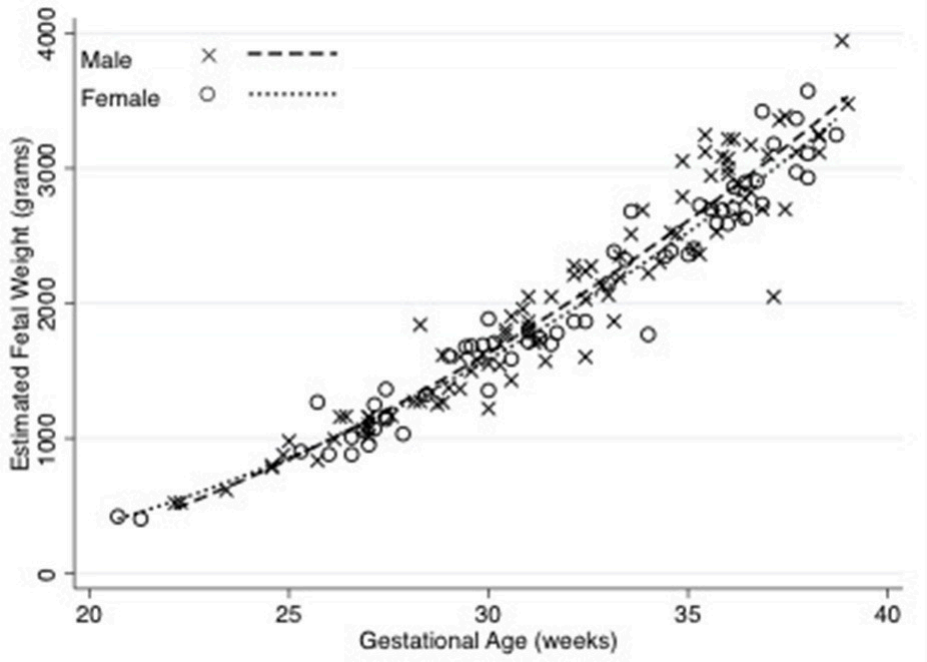
Estimated fetal weight in male and female fetuses across gestation. There was no significant difference in estimated fetal weights of male and female fetuses. The relationship between fetal weight and gestation were derived using non-linear regression. *N* = 100 male and 61 female observations. Equations are: male estimated fetal weight = 868–120.7 (Gestational Age) + 4.86 (Gestational Age^2^) (*R*^2^ = 0.93), female estimated fetal weight = 1143.96–135.22 (Gestational Age) + 4.98 (Gestational Age^2^) (*R*^2^ = 0.95).

The median birth weight for the cohort (including those born prematurely) was 3,300 g, with males weighing 3,357 g and females weighing 3,185 g. The median birth weight of term babies was 3,387 g. Males weighed 3,455 g and females weighed 3,217 g but this difference was not significant. Out of the 24 neonates born prematurely, 18 were male and 6 were female. Thirteen were born to mothers that smoked, while 8 were to non-smokers (three neonates did not have their mother's smoking status recorded). The premature males weighed 2,695 g and females weighed 2,862 g. Estimated fetal weight for male and female fetuses during pregnancy were similar, as shown in Figure 1.

**Figure 2:**
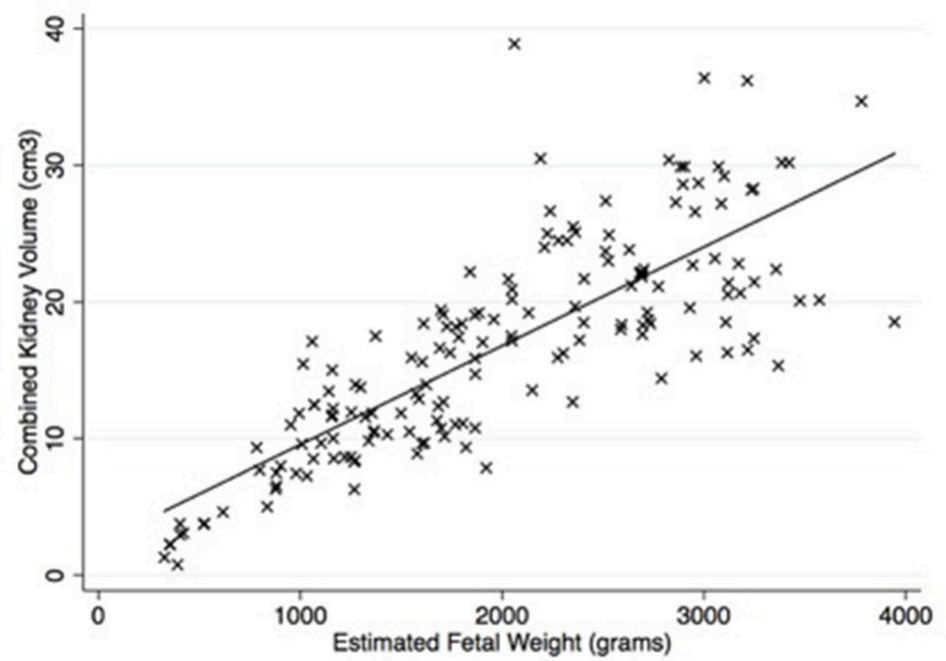
There was a significant correlation between estimated fetal weight and combined kidney volume (rho = 0.82, *p* < 0.001). Equation is *y* = 2.32 + 0.007 (estimated fetal weight). Number of observations = 164.

**Figure 3:**
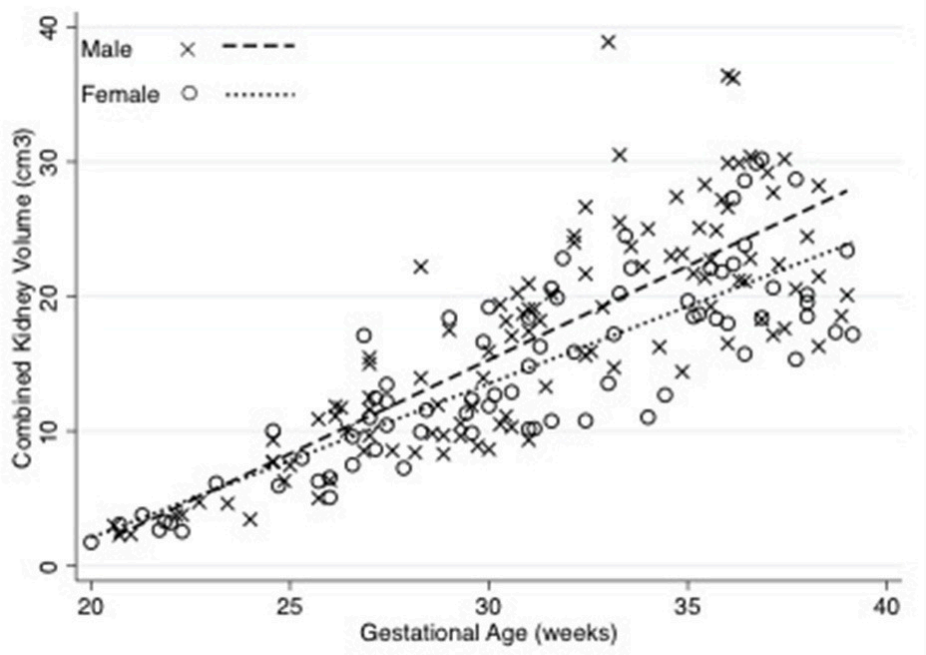
Combined Kidney volume of male and female fetuses throughout gestation. The relationships between fetal kidney volume and gestation were derived using linear regression. The fetal kidney volumes of males were significantly larger than for females (*p* = 0.03). Equations are: Combined kidney volume for males = –23.84 + 1.31 (weeks gestation) (*R*^2^ = 0.70), Combined kidney volume for females = −19.22 + 1.10 (weeks gestation) (*R*^2^ = 0.76). *N* = 117 male and 84 female observations.

Figure 2 shows that there was a strong correlation (spearman's rho 0.82, *p* < 0.001) between estimated fetal weight and combined renal volume. As shown in Figure 3, the fetal kidney volumes of males were significantly larger than for females (*p* = 0.03). Female fetuses also had a smaller fetal kidney volume: body weight ratio than male fetuses [0.38%, compared with 0.44% (*p* = 0.02)].

**Figure 4:**
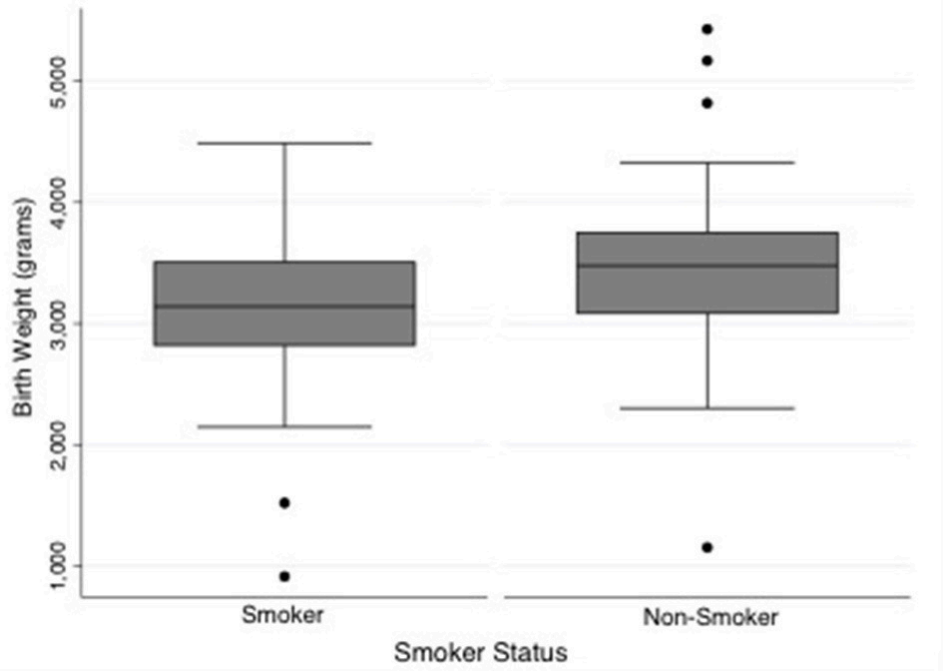
Comparison of birth weight between neonates of smoking and non-smoking mothers. Babies of mothers that smoked were smaller at birth than babies born from non-smoking mothers (*P* = 0.001). Median weight and interquartile range: smoking group = 3,140 g [IQR = 685], non-smoking group = 3,477 g [IQR = 665]. *N* = 61 smokers and 78 non-smokers.

**Figure 5:**
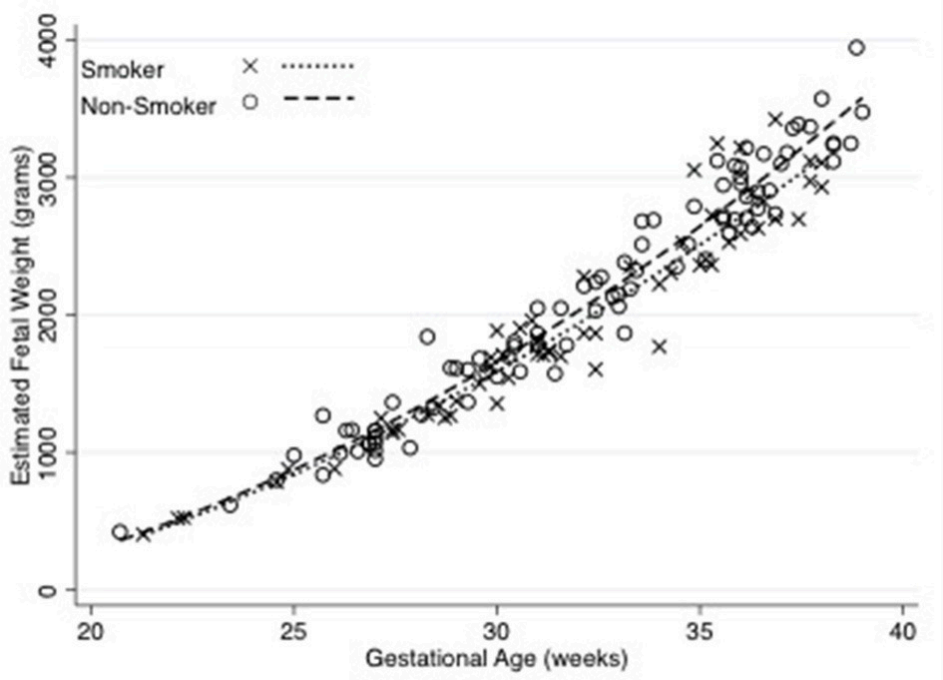
Estimated fetal weight throughout gestation by smoking status of the mother. The relationships between fetal growth and gestation were derived using non-linear regression. Equations are: Smokers = 1165.78–135.23 (Gestational Age) + 4.96 (Gestational Age^2^) (*R*^2^ = 0.93), Non-smokers = −282.37–47.64 (Gestational Age) + 3.75 (Gestational Age^2^) (*R*^2^ = 0.96). Number of observations for smokers = 62 and Non-smokers = 90.

One hundred and thirty-nine out of the 158 mothers completed the smoking survey; 44% reported smoking during pregnancy. The mean gestational age at delivery of the smoking group (38 weeks, IQR 37-39.6) was less than the non-smoking group (39 weeks, IQR 38.4–40.4, *p* = 0.02). Babies of mothers that smoked were smaller at birth (337 g, *P* = 0.001), than babies born from non-smoking mothers (Figure 4). This difference was still apparent when only term babies were considered; that is term babies of smoking mothers were significantly smaller than term babies from non-smoking mothers (327 g, *p* < 0.001). Fetuses of mothers who smoked had lower estimated fetal weights than those who did not smoke (Figure 5). After assessing the effect of smoking at different stages in gestation (20–24, 25–29, 30–34, 35–40 weeks), a significant divergence first occurred at 30–34 weeks (*p* = 0.02). Median birth weights of term male and female fetuses from smoking and non-smoking mothers are shown in Table 1.

**Table 1:** Birth weights (g) of term male and female neonates by smoking status of the mother.

	**Male**	**Female**
Non-smoker	3,520 (520)	3,312 (682)
Smoker	3,357 (597)	2,910 (715)
Difference	163 (*p* = 0.025)	402 (*p* = 0.028)

*Premature neonates were excluded. Data are presented as Median (IQR). N = 32 males and 16 females in the smoker groups, and 39 males and 31 females in the non-smoker groups*.

Term fetuses of mothers who smoked had smaller kidney volumes than those who did not smoke (*p* = 0.02), however, this difference disappeared when sex was taken into account (male *p* = 0.07, female *p* = 0.16). Since the kidney volume to estimated fetal weight ratios were the same in fetuses from smokers and non-smokers, the reduction in renal volumes of fetuses from smokers was proportional to the reduction in birth weight. Assessing individual renal parameters showed that the renal AP diameter was statistically different between the smoking and non-smoking groups (*p* = 0.01). This persisted when males were considered separately (*p* = 0.03), but was not significantly different among females (*p* = 0.15). There was no significant difference between smokers and non-smokers with regard to transverse diameter. Renal length was significantly different overall between the smoking and non-smoking groups, but when analyzed separately for sex, significance was achieved for males (*p* = 0.05), but not for females (*p* = 0.56).

There were no relationships between fetal kidney volumes and maternal urinary protein to creatinine ratio, maternal urinary albumin nor to maternal serum sodium to potassium ratio (data not shown).

## Discussion

We used ultrasound to estimate renal volumes as a surrogate measure of nephron number (Nyengaard and Bendtsen, 1992). There is a significant correlation between renal mass and nephron number, with Zhang et al. reporting an additional 23,459 glomeruli per gram of kidney mass (Zhang et al., 2008). Furthermore, Nyengaard et al. showed that glomerular number correlated with kidney size (Nyengaard and Bendtsen, 1992). Although this association has been questioned (Bueters et al., 2013), use of neonatal renal volumes as a surrogate measure for nephron number has been accepted (Kandasamy et al., 2013, 2014).

Males had larger kidneys both in terms of the total size, and in proportion to body weight. In this study we found that smoking was associated with a reduction in birth weight. The reduction in renal volume was proportional to the reduction in estimated fetal weight.

It is postulated that a reduction in nephron number predisposes to hypertension and renal disease (Brenner et al., 1988; Hinchcliffe et al., 1992; Keller et al., 2003; Hoy et al., 2006; Valerie and Brenner, 2010). Factors that influence nephron number can be classified as genetic or environmental (Hoy et al., 2005). A 10% reduction in kidney size is seen with the inheritance of the RET 1476A polymorphism, as well as with a polymorphic variant of PAX2. When an individual inherits both of these there is a 23% reduction in kidney volume (Zhang et al., 2008). Environmental factors include uteroplacental insufficiency, low protein diet, vitamin A deficiency, hyperglycaemia, cocaine, alcohol and medications such as dexamethasone (Charlton et al., 2014). Low birth weight and intrauterine growth restriction (IUGR) are considered to be an outcome of a suboptimal *in utero* environment. In these settings, the development of the kidneys is vulnerable, leading to reduced nephron number and there is a strong correlation between body weight and glomerular number (Mañalich et al., 2000).

In our cohort, 15.2% of neonates were born prematurely (18 male and 6 female). This is higher than the rate reported by the NSW Ministry of Health (2016), who reported that 12.7% of Indigenous Australian mothers in NSW delivered prematurely in 2015, compared with just 7.7% of non-Indigenous Australian women (Centre for Epidemiology and Evidence, 2016). Premature birth can also affect renal development and nephron number. Nephrogenesis can continue in the context of prematurity, however in autopsied preterm human kidneys and a baboon animal model, it was demonstrated that the cortically located glomeruli, which are formed last, are morphologically abnormal. It is suggested that the abnormal glomeruli are formed in the extra-uterine environment (Sutherland et al., 2011). In the present study we did not determine the impact of prematurity on kidney volume.

In Australia, in 2012, the average birth weight of live born babies was 3,367 g for all mothers. Nationally, babies born to Indigenous mothers were on average 3,211 g. Within NSW the mean birth weight for Aboriginal babies was 3,245 g (Hilder et al., 2012). In our cohort, the median birth weight was 3,300 g, slightly heavier than the national average for Indigenous neonates, which could be influenced by having disproportionally more male neonates in the cohort. In this study, we have shown that fetal weight and kidney volume are related. Hughson et al. (2003) demonstrated a direct relationship between the number of glomeruli within the kidney and birth weight in both adults and fully developed postnatal kidneys of children. They found a direct correlation between birth weight and the number of glomeruli (Hughson et al., 2003).

In 2007–2011, nationally, Indigenous female neonates weighed 125 g less than male babies (3,123 g compared with 3,248 g), which is consistent with our findings (Australian Institute of Health and Welfare, 2014). Others have also found that male neonates have heavier birth weights (Hughson et al., 2003, 2006). Hoy et al. found that nephron number tended to be lower in females than in males, by as much as 11% in a combined ethnic group, and by 5% in Aboriginal people, these findings were not statistically significant (Hoy et al., 2006). Another study involving Aboriginal children aged 5–18 years, also demonstrated lower renal volumes (and thus nephron number) on ultrasound among female participants, which persisted when adjusted for body surface area (Spencer et al., 2001). It is interesting to consider this in the context of data for kidney disease in Australia. In 2007–2008, Indigenous males and females had rates of end-stage renal disease (ESRD) 5–8 times that of non-Indigenous Australians. The incidence of ESRD for Indigenous females was 82.3 patients per 100,000 population, while males were 76.1 per 100,000 (not significantly different Australian Institute of Health and Welfare, 2011.)

In Australia in 2015, 45% of pregnant Indigenous mothers reported smoking during pregnancy, compared with 7.4% of non-Indigenous mothers (Centre for Epidemiology and Evidence, 2016). In our cohort, 44% reported smoking during pregnancy. We showed that neonates of mothers who smoked during pregnancy had lower birth weights than non-smokers. Overall, this reduction in weight in term neonates was around 320 g. This is consistent with broader findings (Conter et al., 1995; Chan and Sullivan, 2008; U.S. Department of Health and Human Services, 2014). This study did not demonstrate any change in kidney volume (when adjusted for estimated fetal weight) in fetuses of mothers who smoked during pregnancy. Others, such as Taal et al. have previously shown changes in fetal kidney volume with maternal smoking, when corrected for estimated fetal weight. In their study they found an increase in kidney volume in the offspring of mothers who smoked less than 5 cigarettes per day, and a reduction in kidney volume in those who smoked more than 10 cigarettes per day (Taal et al., 2011). A limitation of our study was that we did not take into account the number of cigarettes smoked by the mother. The participants were simply classified as smoker or non-smoker. Future studies that either grouped mothers based on the amount of cigarette smoking or to relate kidney volumes to maternal cotinine levels may provide more clarity.

Our data indicates a link between maternal smoking and renal size at birth, which is itself a proxy for nephron number and future risk of hypertension and renal failure. The loss of this association when male and female infants were analyzed separately is probably consequent upon the fewer numbers of infants in each group. Further studies will evaluate the ongoing renal health of these infants to determine their risk of disease. Additionally health promotion activities will continue in these communities to build health literacy surrounding kidney health.

## Conclusion

In a cohort of Indigenous Australians living in rural NSW we showed that maternal smoking was associated with increased risk of preterm birth and independently a reduction in fetal weight with a proportional reduction in renal size. Overall the birth weights were lower than those reported in non–Indigenous Australians, and consistent with the reported national average for Indigenous Australians. This has important implications for Indigenous offspring of low birth weight, as they will also have smaller kidneys (i.e., a reduction in nephron number). Maternal smoking is an important public health issue as it predisposes to reduced renal volume, which has been causally linked to hypertension and renal failure in adult life. This is particularly important to address, given the very high rate of smoking amongst pregnant Indigenous women, whose offspring are already a part of a high-risk group for hypertension and renal failure.

## Author contributions

This paper was the original concept of RS, EL, KP, KR and has been primarily written by CD, KP, EL, and KR. LW, LK: were responsible for recruitment of all participants and the data management for the study; VJ: completed the ultrasound measures on participants. All other authors were involved in the editing and writing of this manuscript and provided critical intellectual comments.

### Conflict of interest statement

The authors declare that the research was conducted in the absence of any commercial or financial relationships that could be construed as a potential conflict of interest. The reviewer KC and handling Editor declared their shared affiliation.
